# Functional role of THRAP3 in modulating thyroid hormone–mediated gene networks in C2C12 myotubes

**DOI:** 10.1371/journal.pone.0341353

**Published:** 2026-01-22

**Authors:** Taku Fukushima, Sachi Kuse, Yuka Hasegawa, Taiju Fujioka, Takeshi Nikawa, Satoru Masubuchi, Iori Sakakibara

**Affiliations:** 1 Department of Physiology, School of Medicine, Aichi Medical University, Nagakute, Aichi, Japan; 2 Department of Nutritional Physiology, Institute of Medical Nutrition, Tokushima University Graduate School, Tokushima, Japan; Kyoto University Graduate School of Medicine Faculty of Medicine: Kyoto Daigaku Daigakuin Igaku Kenkyuka Igakubu, JAPAN

## Abstract

Thyroid hormone (TH) secreted by the thyroid gland plays essential roles in regulating metabolism, development, and nervous system function. Thyroid hormone receptor-associated protein 3 (THRAP3) is a nuclear coactivator that interacts with the thyroid hormone receptor (TR) and facilitates target gene regulation through the mediator complex. Although this mechanism has been well studied in other tissues, the specific role of THRAP3 in skeletal muscle remains unclear. Here we investigated the function of THRAP3 in skeletal muscle using *Thrap3* knockout (KO) C2C12 cells. Loss of THRAP3 significantly suppressed the expression of key myogenic regulatory factors, including *Myod1*, *Mef2c*, and myosin heavy chain genes, resulting in impaired myogenic differentiation and muscle diameter. Furthermore, we found that THRAP3 influences triiodothyronine (T3)-induced gene expression, suggesting that it cooperatively modulates thyroid hormone signaling in muscle cells. Taken together, our findings identify THRAP3 as a novel regulator of myogenesis and indicate that it supports T3 activity by coordinating thyroid hormone–responsive gene expression in skeletal muscle.

## Introduction

Skeletal muscle, accounting for approximately 40% of total body mass, is the largest organ in the body [1]. The maintenance and improvement of muscle function are highly effective strategies for the prevention and treatment of various diseases [2,3]. One of the most remarkable features of skeletal muscle is its regenerative capacity [4,5]. The differentiation process from myoblasts to myotubes is a critical step in skeletal muscle development and regeneration. Myoblast activation and differentiation are tightly regulated by the sequential expression of myogenic regulatory factors (MRFs), including *Myf5*, *Myod1*, *Myogenin* (*Myog*), and *Mrf4*, which coordinate myoblast proliferation, differentiation, and fusion into multinucleated myotubes [6–9]. Therefore, the regulation of MRFs expression plays a critical role in muscle differentiation.

Thyroid hormone (TH) is essential for metabolism, growth, and development, and its dysregulation is implicated in various diseases such as Graves’ disease and Hashimoto’s thyroiditis [10,11]. TH primarily exists in two forms: thyroxine (T4), which is stable in the bloodstream but has low biological activity, and triiodothyronine (T3), the more biologically active form converted from T4 in peripheral tissues [10,12]. TH exerts its function by entering cells and binding to thyroid hormone receptors (TRs), which are members of the nuclear receptor superfamily. Upon binding to TH, TRs activate target genes through recruitment of the mediator complex, one component of which is thyroid hormone receptor-associated protein 3 (THRAP3, TRAP150) [13,14]. THRAP3 is a multifunctional nuclear protein known to regulate transcription by acting as a transcriptional coactivator, modulating RNA polymerase II activity, and influencing circadian rhythms [15–18]. Beyond muscle biology, recent pan-cancer analyses indicate that THRAP3 expression correlates with immune cell infiltration and proliferation/survival pathways in blood cancers [19,20].

Emerging evidence demonstrates that TH modulates muscle satellite cells (MuSCs) function directly [21,22]. For instance, type 3 deiodinase (D3) is highly expressed in activated MuSCs, reducing intracellular T3 to promote satellite cell survival and expansion, while subsequent increases in intracellular T3 via type 2 deiodinase (D2) drive progenitor differentiation [21,23–26]. Additionally, exogenous T3 was shown to enhance myoblast differentiation in C2C12 cells [27], whereas excessive T3 may inhibit proliferation and prematurely induce differentiation, impairing regeneration [21,28]. Thus, a tight control of intracellular T3 levels is essential for MuSCs survival, proliferation, and differentiation. Most recently, TRα was shown to influence muscle cell proliferation and differentiation via aging-related pathway [29]. However, the role of THRAP3 in skeletal muscle is still unclear.

In this study, we utilized a CRISPR/Cas9-based gene knockout (KO) method that we previously established to generate *Thrap3* KO C2C12 myoblasts [30]. Using these KO cells, we investigated the role of THRAP3 during the process of myogenic differentiation. Interestingly, our findings demonstrated that THRAP3 influences myogenesis by regulating key myogenic genes such as *Myod1*, *Mef2c*, and *Myog*. In addition, when we examined the effects of T3 induction under *Thrap3*-deficient conditions, we found that the expression of T3-responsive genes was reduced in the KO group compared to the control (Ctrl) group. Together, these results suggest that *Thrap3* not only regulates gene expression associated with muscle differentiation and formation, but also cooperatively modulates T3-dependent transcriptional responses.

## Materials and methods

### Cell culture

C2C12 cells were kindly provided by Dr. Shinichiro Hayashi. Cells were cultured in high-glucose Dulbecco’s Modified Eagle’s Medium (DMEM) (4.5 mg/mL) (D6429-500ML, Sigma-Aldrich, St. Louis, MO, USA) supplemented with 10% fetal bovine serum (FBS) (10099–141, Thermo Fisher Scientific, Waltham, MA, USA) and 1% penicillin-streptomycin (PS) (26253–84, Nacalai Tesque, Kyoto, Japan). Cultures were maintained at 37°C in 5% CO_2_. When the C2C12 myoblasts were incubated until reaching 80% confluence, cells were seeded at a density of 2 × 10^5^ cells per well in 8-well plates (167064, Thermo Fisher Scientific). To induce myotube differentiation, the medium was replaced with DMEM containing 2% horse serum (HS) (16050–122, Thermo Fisher Scientific) and 1% PS after two days. The differentiation medium was changed every 48 hours. C2C12 cells were treated with 10 nM triiodothyronine (T3) (038–25541, FUJIFILM Wako Pure Chemical, Osaka, Japan) for 24 hours on day 6 of differentiation.

### Immunofluorescence staining

C2C12 myotubes at 6 days post-differentiation were fixed with 4% paraformaldehyde-phosphate buffer in phosphate-buffered saline (PBS; 09154–85, Nacalai Tesque) for 10 min and subsequently permeabilized with PBS containing 1% Triton at room temperature. Fixed cells were washed three times with PBS, with each wash lasting 5 min. Blocking was performed using PBS containing 1% Triton and 2% HS at room temperature for 30 min. Primary antibodies were diluted in blocking buffer and applied as follows: anti-THRAP3 antibody (A9396, ABclonal, Woburn, MA, USA, 1:100) and anti-Myosin Heavy Chain (MyHC) antibody (MF20 clone, MAB4470, R&D Systems, Minneapolis, MN, USA, 1:100). Samples were incubated overnight at 4°C. After primary antibody incubation, cells were washed and then incubated with secondary antibodies: anti-mouse IgG 555 (A28180, Thermo Fisher Scientific, 1:1000) for MyHC and anti-rabbit IgG 448 (A21206, Thermo Fisher Scientific, 1:1000) for THRAP3. Secondary antibody incubation was performed in the dark at room temperature for 1 hour. Nuclei were counterstained with DAPI (340–07971, FUJIFILM Wako Pure Chemical). Samples were visualized using a fluorescence microscope (BZ-X800, KEYENCE, Osaka, Japan) at 10 × magnifications.

### Plasmid construction

Distinct guide RNAs (gRNAs) targeting mouse *Thrap3* gene were designed using the CHOPCHOP [31] website. four pGuide-it-ZsGreen plasmids were constructed by Each pair of oligonucleotides (*Thrap3* gRNA1 sense and *Thrap3* gRNA1 antisense, *Thrap3* gRNA2 sense and *Thrap3* gRNA2 antisense, *Thrap3* gRNA3 sense and *Thrap3* gRNA3 antisense, *Thrap3* gRNA4 sense and *Thrap3* gRNA4 antisense, *Thrap3*#2 gRNA1 sense and *Thrap3*#2 gRNA1 antisense, *Thrap3*#2 gRNA2 sense and *Thrap3*#2 gRNA2 antisense, *Thrap3*#2 gRNA3 sense and *Thrap3*#2 gRNA3 antisense, *Thrap3*#2 gRNA4 sense and *Thrap3*#2 gRNA4 antisense, and Negative Control (NC) gRNA sense and NC gRNA antisense) was annealed, then inserted into pGuide-it-ZsGreen vector (Z2601N, TaKaRa, Shiga, Japan) according to the manufacturer’s instructions. The plasmids were named as pGuide-it-ZsGreen-*Thrap3*_1, pGuide-it-ZsGreen-*Thrap3*_2, pGuide-it-ZsGreen-*Thrap3*_3, pGuide-it-ZsGreen-*Thrap3*_4, pGuide-it-ZsGreen-*Thrap3*#2_1, pGuide-it-ZsGreen-*Thrap3*#2_2, pGuide-it-ZsGreen-*Thrap3*#2_3, pGuide-it-ZsGreen-*Thrap3*#2_4, and pGuide-it-ZsGreen-NC, respectively. The sequences of the inserted oligonucleotides for *Thrap3* KO cell (KO) were as follows; *Thrap3* gRNA1 sense: CCG GAA ACG AAC GAG ACC GAG ATC; *Thrap3* gRNA1 antisense: AAA CGA TCT CGG TCT CGT TCG TTT; *Thrap3* gRNA2 sense: CCG GGT CGG GGC CGT TCT CGA TCC; *Thrap3* gRNA2 antisense: AAA CGG ATC GAG AAC GGC CCC GAC; *Thrap3* gRNA3 sense: CCG GCG GTT CTG CAT CAC GGG CCT; *Thrap3* gRNA3 antisense: AAA CAG GCC CGT GAT GCA GAA CCG; *Thrap3* gRNA4 sense: CCG GTT TCC GGG TGA CTG CGT ACA; *Thrap3* gRNA4 antisense: AAA CTG TAC GCA GTC ACC CGG AAA; Negative Control (NC) gRNA sense: CCG GTG AGA CGA AAA ACG TCT CA; NC gRNA antisense: AAA CTG AGA CGT TTT TCG TCT CA. And the sequences of the inserted oligonucleotides for another *Thrap3* KO cell (KO#2) were as follows; *Thrap3*#2 gRNA1 sense: GCC GAT CTG TCT CTC GTT CA; *Thrap3*#2 gRNA1 antisense: TGA ACG AGA GAC AGA TCG GC; *Thrap3*#2 gRNA2 sense: GAC GAG GGC TGT ATG CTT GC; *Thrap3*#2 gRNA2 antisense: GCA AGC ATA CAG CCC TCG TC; *Thrap3*#2 gRNA3 sense: ATG GCG CCG GTT CTG CAT CA; *Thrap3*#2 gRNA3 antisense: TGA TGC AGA ACC GGC GCC AT; *Thrap3*#2 gRNA4 sense: GGA GCG CTC CTC ACC GTG CA; *Thrap3*#2 gRNA4 antisense: TGC ACG GTG AGG AGC GCT CC. And the sequences of the inserted oligonucleotides for Negative Control (NC) gRNA sense: CCG GTG AGA CGA AAA ACG TCT CA; NC gRNA antisense: AAA CTG AGA CGT TTT TCG TCT CA. All plasmid sequences were verified by sequencing.

### Generation of *Thrap3*-Knockout (KO) C2C12 cells

The overall workflow and experimental procedures for generation of knockout cells were previously described in our study [30]. To generate *Thrap3*-deficient C2C12 cells, four plasmids (pGuide-it-ZsGreen-*Thrap3*_1, pGuide-it-ZsGreen-*Thrap3*_2, pGuide-it-ZsGreen-*Thrap3*_3 and pGuide-it-ZsGreen-*Thrap3*_4) were pooled and co-transfected into C2C12 myoblasts. In addition, an independent knockout line (KO#2) was generated using the other four plasmid (pGuide-it-ZsGreen-*Thrap3*#2_1, pGuide-it-ZsGreen-*Thrap3*#2_2, pGuide-it-ZsGreen-*Thrap3*#2_3 and pGuide-it-ZsGreen-*Thrap3*#2_4). As a control (Ctrl), C2C12 cells were transfected with pGuide-it-ZsGreen-NC. Transfection of all plasmids was performed using jetPRIME reagent (101000027, Polyplus, Illkirch-Graffenstaden, France), according to the manufacturer’s instructions. Briefly, C2C12 cells were seeded at a density of 5 × 10^4^ cells/well in 6-well plates containing DMEM supplemented with 10% FBS for 24 hours prior to transfection. The plasmid pool (500ng of each gRNA plasmid) or the control plasmid was then transfected into the cells using jetPRIME.

At 24 hours after transfection, ZsGreen fluorescence was confirmed by fluorescence microscopy observation. ZsGreen-positive cells were then isolated using a cell sorter (SH800, Sony, Tokyo, Japan). The sorted cells were subcultured, expanded for 6 days, and subsequently cryopreserved at −80°C.

### Western blot analysis

C2C12 myotubes at 6 days post-differentiation were homogenized in lysis buffer, followed by centrifugation. Protein concentrations were measured using a Protein Assay BCA Kit (297−73101, FUJIFILM Wako Pure Chemical). Protein samples were separated by 4−15% Mimi-PROTEAN TGX Precast Protein Gels (4561086, Bio-Rad, Hercules, CA, USA) and transferred to PVDF membranes. Primary antibodies used were anti-THRAP3 (A9396, ABclonal, 1:1000) and anti-β-Actin (010−27841, FUJIFILM Wako Pure Chemical, 1:1000). Secondary antibody included anti-rabbit IgG-HRP (SA00001−2, Proteintech, IL, USA, 1:10000) for THRAP3 detection and anti-mouse IgG-HRP (SA00001−1, Proteintech, 1:10000) for β-Actin detection. Protein bands were visualized using ImageQuant LAS 500 (Cytiva, Tokyo, Japan). Quantitative analysis was performed using ImageJ software.

### RNA extraction

Cryopreserved *Thrrap3* control (Ctrl) and knockout (KO) cells were differentiated into myotubes, and total RNA was extracted at 0-, 2-, 4-, and 6-days post differentiation using RNAiso Plus Reagent (9109, TaKaRa) according to the manufacturer’s instructions. RNA concentration was quantified using a Nanodrop 1000 Spectrophotometer (Thermo Fisher Scientific). RNA purity was assessed using the 260 nm/280 nm absorbance ratio. The extracted RNA samples were stored at −80°C.

### Quantitative real-time PCR analysis (qRT-PCR)

Reverse transcription of total RNA (500 ng) was performed using PrimeScript RT Master Mix (RR036A, TaKaRa). qRT-PCR was conducted in a final volume of 10 µl using GeneAce SYBR qPCR Mix II (313–09423, NIPPON GENE, Tokyo, Japan) according to the manufacturer’s instructions. All reactions were performed on a StepOne Real-Time PCR System (Applied Biosystems, Waltham, MA, USA). The sequences of primers used in this study are listed in Table 1. Beta-actin (*Actb*) was used as an internal control for normalization.

**Table 1:** The sequence of the oligonucleotides for qPCR.

Gene name	Forward (5’- 3’)	Reverse (5’- 3’)
*Actb*	GGCTGTATTCCCCTCCATCG	CCAGTTGGTAACAATGCCATGT
*Myh7*	AGGGCGACCTCAACGAGAT	CAGCAGACTCTGGAGGCTCTT
*Myh2*	ACTTTGGCACTACGGGGAAAC	CAGCAGCATTTCGATCAGCTC
*Myh4*	GCTTGAAAACGAGGTGGAAA	CCTCCTCAGCCTGTCTCTTG
*Myh1*	TACTCACGCCAGCTAGACGA	TGCCTCTTCAGCTCCTCAAT
*Myod1*	TACCCAAGGTGGAGATCCTG	CATCATGCCATCAGAGCAGT
*Myog*	GAGACATCCCCCTATTTCTACCA	GCTCAGTCCGCTCATAGCC
*Mef2c*	GTCAGTTGGGAGCTTGCACTA	CGGTCTCTAGGAGGAGAAACA
*Ppargc1a*	TATGGAGTGACATAGAGTGTGCT	CCACTTCAATCCACCCAGAAAG
*Mybpc2*	ATGCCTGAGGCTAAACCAGC	ACACAGAGTCCGGCTTTTTCA
*Thrap3*	TCTCGGTCTCGTTCGTTTTCA	TCCTTTCTCTGTTATGAGCTGGA
*Cox6c*	GCGTCTGCGGGTTCATATTG	TCTGCATACGCCTTCTTTCTTG
*Ndufs2*	TTTCGGGAGCTGTCATGTACC	TGGTCACCGCTTTTTCCTTCA
*mt-Co3*	CCAAGGCCACCACACTCCTA	GGTCAGCAGCCTCCTAGATCA
*mt-Atp6*	AGCTCACTTGCCCACTTCCT	AAGCCGGACTGCTAATGCCA
*mt-Nd3*	TAGTTGCATTCTGACTCCCCCA	GAGAATGGTAGACGTGCAGAGC
*mt-Nd4*	CGCCTACTCCTCAGTTAGCCA	TGATGTGAGGCCATGTGCGA
*Xirp1*	GCTCCGGCGTCTCTACAAAC	CCAGCGCATACACTGAACATC
*Mybph*	CCTGAACCTCCGAGTGAAGAT	TCCAACACATAGCCTTGAAGC

### RNA-sequencing (RNA-seq) and bioinformatics analysis

Total RNAs extracted from C2C12 cells at day 7 post-differentiation were used for RNA-sequencing (RNA-seq). Libraries were prepared using NEBNext® Ultra™II Directional RNA Library Prep Kit (New England Biolabs, MA, USA) according to the manufacturer’s instructions. Sequencing was performed on a DNBSEQ-T7 device (MGI, Shenzhen, China). Using the RaNA-seq framework, the sequenced reads were mapped to the mouse reference genome (GRCm38). Hierarchical clustering analysis of the data was performed as previously described [32]. RNA-seq data were deposited in the Gene Expression Omnibus (accession GSE306461). Gene ontology (GO) enrichment, Kyoto encyclopedia of Genes and Genomes (KEGG) pathway, and BP DIRECT analyses were conducted using the ShinyGO (version 0.81) tool [33]. Hierarchically clustered heatmaps were analyzed using Morpheus software (https://software.broadinstitute.org/morpheus).

### Cell proliferation assay

C2C12 myoblasts were cultured in DMEM supplemented with 10% FBS and 1% PS at 37°C in a humidified atmosphere containing 5% CO₂. For the cell proliferation assay, cells were seeded at a density of 5 × 10³ cells per well in 96-well plates (92696, TPP, Switzerland) and analyzed at 0 h, 24 h, and 48 h after seeding. Cell proliferation was evaluated using the MTT Cell Count Kit (23506−80, Nacalai Tesque) according to the manufacturer’s instructions, and absorbance was measured at 570 nm using a SpectraMax ABS Plus microplate reader (Molecular Devices, San Jose, CA, USA).

### Public database analysis

Public RNA-seq data from the Adult Genotype Tissue Expression (GTEx) were used to evaluate THRAP3 expression across human tissues. The data used for the analyses described in this study were obtained from the GTEx Portal (https://gtexportal.org) on 10/17/2025, and additional data were accessed from dbGaP accession number phs000424.v2.p1 on 10/17/2025, in accordance with GTEx usage guidelines. Expression values (TPM) for THRAP3 (ENSG00000185022.12) were downloaded and visualized to compare its expression levels among major tissues, including skeletal muscle.

Publicly available microarray data from the NCBI Gene Expression Omnibus (GEO) dataset GSE469 [34] were downloaded on 10/17/2025 and analyzed to assess *Thrap3* expression during muscle regeneration.

### Statistical analysis

Results are expressed as mean ± standard deviation (SD). Comparisons between two groups were performed using unpaired Student’s *t*-tests. For comparisons among multiple groups, one-way analysis of variance (ANOVA) followed by Tukey’s honestly significant difference (HSD) post hoc test was used. Statistical significance is denoted as follows: *p < 0.05; **p < 0.01; ***p < 0.001; ****p < 0.0001.

## Results

### THRAP3 expression in human skeletal muscle and C2C12 cells

**Fig 1:**
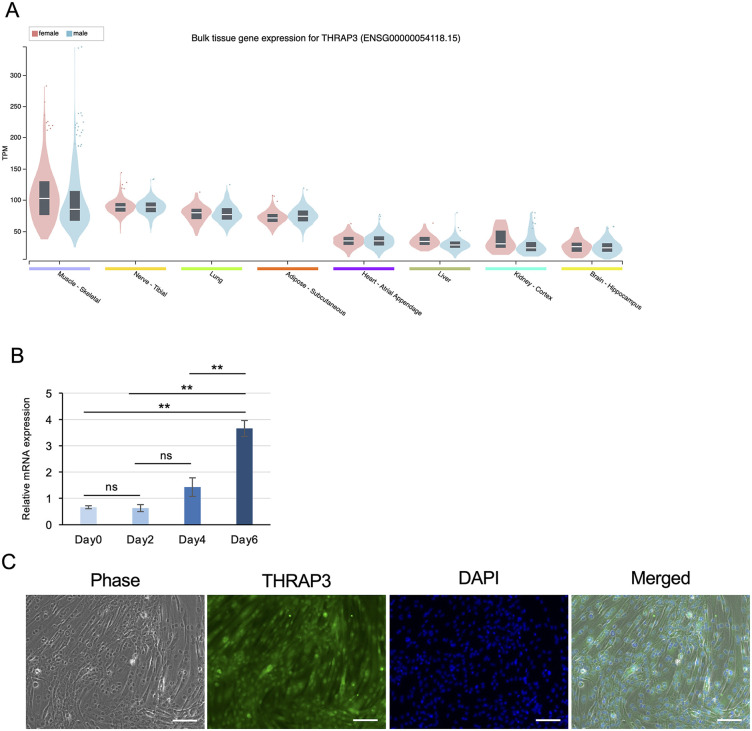
*Thrap3* expression and THRAP3 localization in human skeletal muscle and C2C12 myocytes. **(A)** *THRAP3* expression across human tissues based on GTEx bulk RNA-seq (TPM). Violin plots show THRAP3 expression in male (blue) and female (pink) samples. **(B)** *Thrap3* mRNA expression levels during C2C12 myoblast differentiation (n = 4/group). **(C)** Immunofluorescence staining of THRAP3 (green) and nuclei (DAPI, blue) in C2C12 myotubes on day 6 post-differentiation. Scale bar, 100 μm.

Using GTEx bulk RNA-seq data, we examined *THRAP3* expression across multiple human tissues. *THRAP3* was expressed in all surveyed tissues; however, its expression was particularly elevated in skeletal muscle compared with other major organs (Fig 1A). Violin plot analysis demonstrated that both male and female skeletal muscle samples exhibited consistently higher TPM values than other tissues. We also examined *Thrap3* expression in mice skeletal muscle during muscle regeneration following cardiotoxin-induced injury, showing *Thrap3* is constantly expressed during muscle regeneration (S1 Fig). These findings indicate that THRAP3 is not only ubiquitously expressed but also enriched in skeletal muscle, supporting its potential importance in muscle-specific gene regulatory processes.

Next, we analyzed *Thrap3* mRNA in C2C12 myogenic cells. To examine the temporal expression pattern during myogenesis, we measured *Thrap3* mRNA levels in differentiating C2C12 myocytes. Expression gradually increased from day 0 to day 6 post-differentiation, reaching maximal levels at day 6 (Fig 1B).

Immunofluorescence analysis on day 6 post-differentiation revealed that THRAP3 protein was localized in both the nucleus and cytoplasm of differentiated myotubes (Fig 1C). Collectively, these results indicate that *Thrap3* is more highly expressed in myotubes than in myoblasts and that THRAP3 protein exhibits both nuclear and cytoplasmic localization.

### Generation and characterization of *Thrap3* knockout C2C12 cells

**Fig 2:**
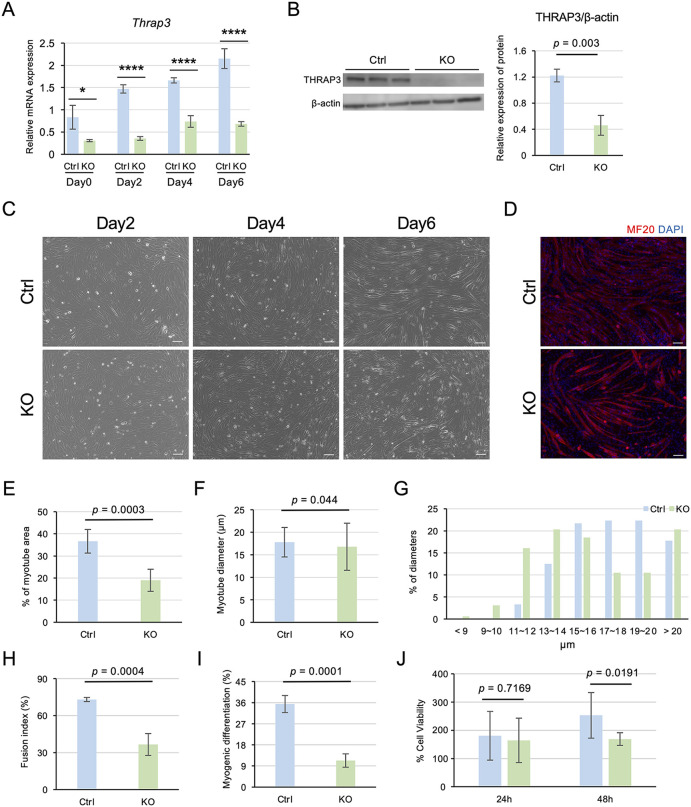
Function analysis of *Thrap3* knockout (KO) C2C12 cell. (A) mRNA expression levels of *Thrap3* in control (Ctrl) and knockout (KO) C2C12 cells during differentiation (n = 4/group). **(B)** Representative western blot and quantification of THRAP3 protein expression in Ctrl and KO cells at day 6 post-differentiation (n = 3/group). **(C)** Phase-contrast microscopy images showing morphological changes in Ctrl and KO cells at days 2, 4, and 6 post-differentiation. Scale bar, 100 μm. **(D)** Immunofluorescence staining of Myosin Heavy Chain (MyHC, red) and nuclei (blue) in Ctrl and KO cells at day 6 post-differentiation. Scale bar, 100 μm. **(E)** Quantification of total MyHC-expressing area in Ctrl and KO cells at day 6 post-differentiation (n = 6/group). **(F)** Average myotube diameter in Ctrl and KO cells at day 6 post-differentiation (Ctrl, n = 152; KO, n = 162). **(G)** Distribution of myotube diameter in Ctrl and KO cells at day 6 post-differentiation (Ctrl, n = 152; KO, n = 162). **(H)** Fusion index, defined as the ratio of MyHC-positive nuclei to the total nuclei within multinucleated myotubes, in Ctrl and KO cells at day 6 post-differentiation (n = 4/group). **(I)** Myogenic differentiation, defined as the percentage of MyHC-positive cells among all myotubes, in Ctrl and KO cells at day 6 post-differentiation (n = 4/group). **(J)** Cell viability rate in Ctrl and KO cells at 24 and 48 hours, as assessed by the MTT assay. (n = 8/group).

To assess the relevance of THRAP3 in muscle function, we generated *Thrap3* knockout (KO) C2C12 myoblasts using the CRISPR/Cas9-based genome editing system established in our previous study [30]. Compared to the Ctrl group, the KO group exhibited a stable and significant reduction in *Thrap3* mRNA expression levels (Fig 2A). Similarly, THRAP3 protein levels at day 6 post-differentiation were significantly suppressed in the KO group (Fig 2B).

Morphological changes during differentiation were monitored from day 2 to day 6 in both cell lines (Fig 2C). To evaluate the impact of *Thrap3* deficiency on myotube formation, immunofluorescence staining was performed using anti-myosin heavy chain antibody (MF20) at day 6 post-differentiation (Fig 2D). Quantitative analysis revealed that myotubes in the KO group exhibited significantly reduced area of myofibers and decreased diameter compared to the Ctrl group (Figs 2E-2G). Furthermore, both the fusion index and the proportion of myogenic differentiation were significantly reduced in the KO group compared with the Ctrl group (Figs 2H and 2I). In addition, we evaluated cell proliferation by a MTT assay, no significant difference was observed between the two groups at 24 hours post-seeding. However, by 48 hours, cell viability was significantly decreased in the KO group relative to the Ctrl group (Fig 2J). These results suggest that *Thrap3* deficiency impairs myotube formation and may play a critical role in myogenesis.

### Gene expression dynamics in *Thrap3* KO cells during myogenic differentiation

**Fig 3:**
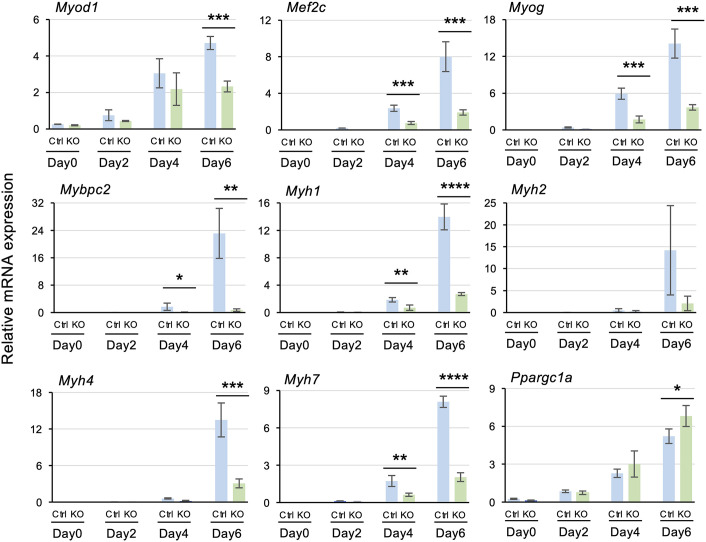
Effects of *Thrap3* deletion on myogenesis-related gene expression. mRNA expression levels of *Myod1*, *Mef2c*, *Myog*, *Mybpc2*, *Myh1*, *Myh2*, *Myh4*, *Myh7*, and *Ppargc1a* in Ctrl and KO cells, as determined by qRT-PCR (n = 4/group).

Since skeletal muscle undergoes dynamic changes in marker gene expression during differentiation, we examined gene expression profiles in *Thrap3* KO cells from pre-differentiation through day 6 post-differentiation using qRT-PCR. Our analysis revealed that expression levels of key myogenic regulatory genes, including *Myod1*, *Mef2c*, *Myog*, and *Mybpc2*, were significantly reduced in *Thrap3* KO cells compared to Ctrl cells at day 6. Furthermore, muscle structural genes, including *Myh1*, *Myh4*, and *Myh7,* were also significantly downregulated in the *Thrap3* KO group compared to the Ctrl group. Interestingly, *Ppargc1a*, a gene involved in mitochondrial biogenesis, showed significantly elevated expression in *Thrap3* KO cells compared to Ctrl cells at day 6 (Fig 3). However, mitochondria-related genes (*Cox6c*, *Ndufs2*), and mitochondrial genes showed no substantial differences between Ctrl and KO groups (S2 Fig). These results suggest that THRAP3 functions to promote the expression of myogenic marker genes.

To exclude the possibility that these phenotypes resulted from off-target effects of CRISPR/Cas9 editing, we generated another *Thrpa3*-KO cell line (KO#2) by different guide RNAs targeting a different region of *Thrap3*. Western blot analysis confirmed that THRAP3 protein levels were markedly reduced in KO#2 cell line at day 6, consistent with the decrease in *Thrap3* mRNA (S3A and S3B Figs). Moreover, representative *Thrap3*-dependent genes showed similarly reduced expression in the KO#2 cells (S3C Fig). These results support that the observed changes in gene expression were specifically associated with the loss of *Thrap3*.

### RNA-seq analysis reveals THRAP3’s role in regulating myogenesis and oxidative phosphorylation pathways

**Fig 4:**
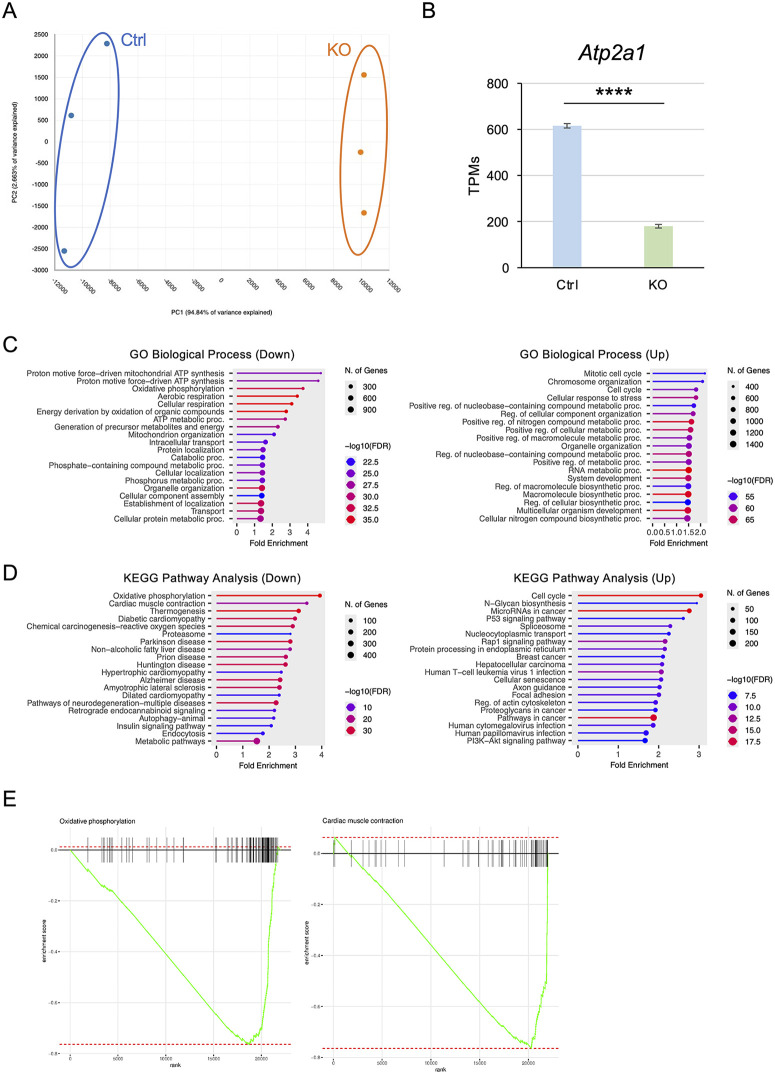
RNA-seq analysis of *Thrap3* KO cells at 7 days post-differentiation. **(A)** PCA with RNA-seq analysis of Ctrl and KO cells (n = 3/group). **(B)** TPM values of *Atp2a1* obtained from RNA-seq analysis (n = 3/group). **(C)** GO biological analysis of differentially expressed genes (DEGs) in *Thrap3* KO C2C12 cells. **(D)** KEGG pathway analysis of DEGs in *Thrap3* KO C2C12 cells. **(E)** GSEA analysis of gene sets related to oxidative phosphorylation and cardiac muscle contraction in *Thrap3* KO C2C12 cells.

To further investigate the role of *Thrap3* in skeletal muscle, we performed RNA-seq analysis of *Thrap3* Ctrl and KO C2C12 cells at day 7 of differentiation. Principal component analysis (PCA) revealed that PC1 accounted for 94.8% of the total variance, indicating that the major source of variation was the difference between the *Thrap3* Ctrl and KO conditions (Fig 4A). *Atp2a1,* previously reported as a target of TRα[27], was also decreased in *Thrap3* KO cells (Fig 4B). We next identified genes significantly affected by *Thrap3* KO (padj < 0.05) and performed gene ontology (GO) analysis, which revealed that 4,018 genes were downregulated and 4,491 genes were upregulated. GO biological process analysis showed that genes related to ATP synthesis and oxidative phosphorylation pathways were significantly downregulated in the *Thrap3* KO group compared to the Ctrl group (Fig 4C). Kyoto encyclopedia of Genes and Genomes (KEGG) pathway analysis further demonstrated that genes involved in oxidative phosphorylation and cardiac muscle contraction pathways were also suppressed in the KO group relative to the Ctrl group (Fig 4D). To determine whether these pathways were systematically biased in the expression data, we conducted a gene set enrichment analysis (GSEA), which confirmed that gene sets related to oxidative phosphorylation and cardiac muscle contraction were downregulated in the KO group (Fig 4E).

We then extracted the top 100 differentially expressed genes with the largest changes. Among these, genes related to myosin formation (*Myh1*, *Myh4*, *Myh8*, *Myl1*, *Myl2*, *Mylpf*, *Myl4*, *Myl6b*, *Mybph*), calcium regulation networks (*Ryr1*, *Cacna1s*, *Casq1*, *Casq2*, *Hrc*, *Atp2a1*), and neuromuscular junction (NMJ) formation networks (*Chrna1*, *Chrnd*, *Chrng*, *Scn4a*, *Cacnb1*) were notably downregulated in the KO group (S4 Fig). In addition, genes related to energy metabolism pathways, such as *Ckm*, *Pfkm*, and *Pygm*, were suppressed in the KO group (S4 Fig). Taken together, these findings suggest that THRAP3 contributes to the regulation of gene expression related to NMJ formation, calcium handling, and myogenesis in skeletal muscle.

Furthermore, because reduced cell viability was observed in KO cells (Fig 2J), we performed Gene Ontology analysis of the RNA-seq data to identify genes involved in cell proliferation. This analysis revealed significant downregulation of gene clusters related to muscle organ development, skeletal muscle tissue development, and regulation of the cell cycle in the KO group (S5 Fig).

### T3 treatment induces distinct transcriptional changes in *Thrap3* Ctrl and KO C2C12 myotubes

**Fig 5:**
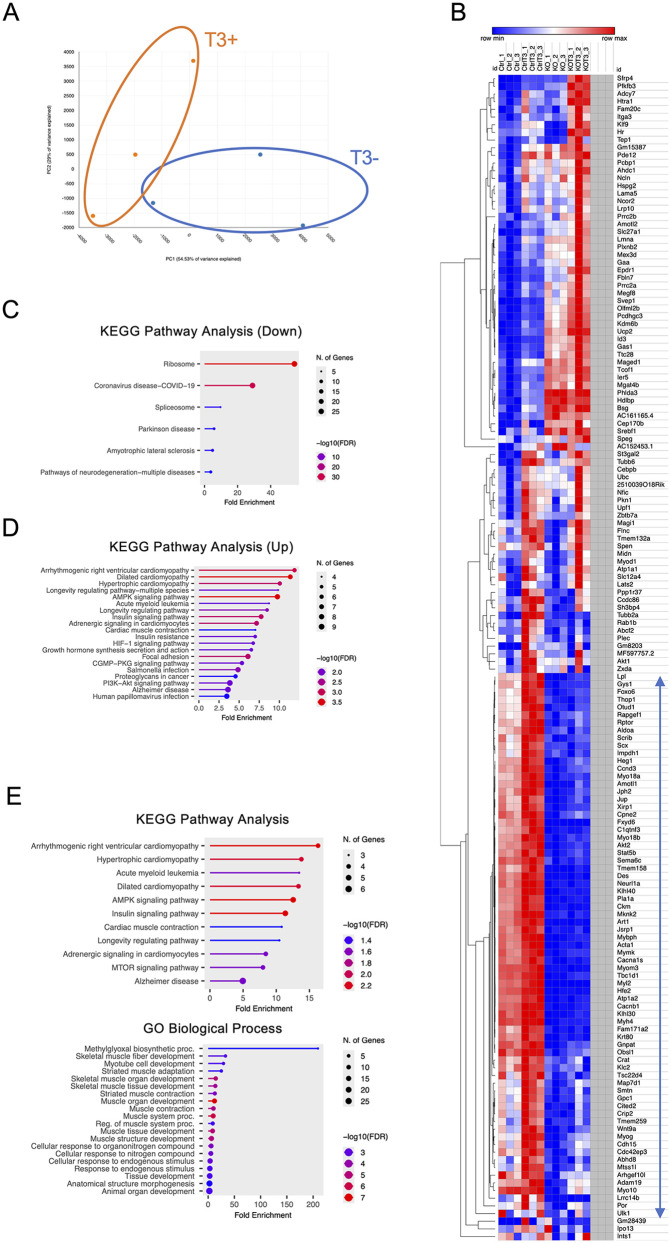
RNA-seq analysis of T3-induced gene expression in *Thrap3* KO cells. **(A)** PCA of RNA-seq data from *Thrap3* Ctrl cells with (T3+) and without (T3-) T3 treatment (n = 3/group). **(B)** Heatmap showing genes significantly upregulated by T3 treatment in the Ctrl group. **(C)** KEGG pathway analysis of DEGs downregulated by T3 addition in *Thrap3* Ctrl C2C12 cells. **(D)** KEGG pathway analysis of DEGs upregulated by T3 addition in *Thrap3* Ctrl C2C12 cells. **(E)** KEGG pathway analysis and GO biological process analysis of the gene cluster spanning from *Lpl* to *Ulk1* in the heatmap.

To investigate how T3 influences gene expression in skeletal muscle cells, we performed RNA-seq analysis of *Thrap3* Ctrl and KO C2C12 cells treated with T3 (10 nM) for 24 hours on day 6 of differentiation. PCA showed a partial separation between T3-treated (T3+) and untreated (T3-) samples in both Ctrl and KO groups (Fig 5A and S6A Fig). Although the clusters were not completely distinct, the overall distribution suggests that T3 treatment exerts transcriptome effects in both genotypes.

To identify T3-responsive genes specifically in the Ctrl group, we generated a heatmap of differentially expressed genes (DEGs) that were significantly upregulated by T3 treatment (padj < 0.05; Ctrl vs Ctrl_T3) (Fig 5B). Additionally, to gain further insights into the biological pathways involved, we conducted KEGG pathway enrichment analysis of DEGs. In Ctrl cells, downregulated genes were primarily enriched in ribosome and spliceosome pathways (Fig 5C), whereas upregulated genes were associated with cardiac muscle contraction, PI3K-Akt signaling, and hypertrophic cardiomyopathy (Fig 5D). In contrast, the pathway profiles in KO cells were markedly different from those in Ctrl cells (S6B and S6C Figs).

To further examine genes induced by T3 but unchanged in the *Thrap3* KO group, we conducted GO analysis on the gene cluster spanning from *Lpl* to *Ulk1* (indicated by the arrows in the heatmap). Enrichment analysis confirmed that these T3-induced genes in the Ctrl group were significantly involved in pathways related to cardiac muscle contraction, AMPK signaling, and insulin signaling (KEGG), as well as GO biological processes including skeletal muscle fiber development, myotube differentiation, and muscle contraction (Fig 5E). To exclude the possibility of off-target effects, we also performed T3 treatment on KO#2 cells and performed qRT-PCR analysis, which produced results consistent with those obtained from the original KO line (S7 Fig).

Together, these results suggest that *Thrap3* plays a key role in modulating the transcriptional response to T3 in C2C12 myotubes, particularly by regulating the induction of genes associated with muscle development and metabolic pathways.

## Discussion

The findings presented here demonstrated that THRAP3 plays an important role in regulating gene expression related to myogenic differentiation, NMJ formation, calcium handling, and energy metabolism in C2C12 myotubes. We found that the loss of THRAP3 significantly impaired the expression of key myogenic regulatory factors such as *Myod1*, *Mef2c*, and *Myog* (Fig 3). In addition, RNA-seq and GO analyses revealed that genes involved in oxidative phosphorylation and cardiac muscle contraction were downregulated in the *Thrap3* KO cells (Fig 4). Notably, T3-induced gene expression was partially diminished under *Thrap3*-deficient conditions, indicating that THRAP3 cooperatively modulates thyroid hormone–mediated transcriptional responses (Fig 5). These findings highlight the multifaceted role of THRAP3 in coordinating skeletal muscle gene networks critical for muscle development and metabolic function.

There is growing evidence that THRAP3 is a multifunctional protein with roles in transcriptional regulation, protein complex formation, and maintaining genome stability through the regulation of R-loop resolution and DNA damage response [35–38]. Although these functions have been reported in cancer cells, adipose tissue, chondrogenesis, and osteogenesis, its role in skeletal muscle remains poorly understood. THRAP3 is also known to interact with TRα[13,14]. Milanesi et al. demonstrated that TRα contributes to myoblast proliferation and differentiation via the regulation of *Atp2a1* (*Serca1*) [27]. Consistently, our RNA-seq data showed significantly reduced *Atp2a1* expression in *Thrap3* KO cells (Fig 4B), supporting the idea that THRAP3 may act as a complex with TRα. In contrast, Shi et al. reported that *Col6a1* was affected during aging [29]. However, we did not observe significant changes in *Col6a1* expression in our dataset. This discrepancy may reflect differences between *in vitro* and *in vivo* aged muscle conditions. *Thrap3* KO cells showed impaired myogenesis and decreased myotube diameter (Fig 2). The reduction of diameter was subtle compared with impaired myogenesis. These results suggested that *Thrap3* regulates myogenesis rather than hypertrophy of myotubes.

Although the direct target genes of THRAP3 in skeletal muscle could not be identified in this study, a previous study provides important mechanistic insight. ChIP-seq data reported by Marcheva et al. demonstrated that THRAP3 is enriched at the promoter region of *Bach1*, indicating that *Bach1* may be a direct transcriptional target of THRAP3 [39]. Consistent with this possibility, our RNA-seq data revealed that *Bach1* expression was reduced in *Thrap3*-KO cells (S8 Fig), further supporting a regulatory relationship between THRAP3 and *Bach1*. Importantly, Patsalos et al. showed that BACH1 modulates skeletal muscle regeneration by regulating macrophage dynamics during injury [40], and Suzuki et al. reported that Bach1 promotes muscle regeneration by suppressing Smad-mediated inhibitory signaling [41]. Together, these findings raise the possibility that THRAP3 might influence myogenic differentiation and muscle regeneration, at least in part, through pathways involving *Bach1*.

Here, our analysis showed that the expression of genes related to E-C coupling—including those involved in the NMJ, calcium handling, and sarcomere structural proteins—was significantly suppressed in the *Thrap3* KO group. E-C coupling is the fundamental mechanism by which action potentials from motor neurons trigger muscle contraction through a coordinated sequence from membrane depolarization to calcium binding to troponin, ultimately leading to contraction [42–44]. *Thrap3* deficiency downregulated genes associated with each of these mechanisms, suggesting that *Thrap3* may critically impact muscle contractile function (S9 Fig). Moreover, we observed that myogenesis-related genes were also affected, raising the possibility that impaired differentiation may secondarily influence the expression of E-C coupling-related genes. However, we did not perform physiological or functional experiments, such as measurements of maximal contractile force or muscle endurance, in this study. Therefore, future studies using skeletal-muscle specific knockout mice will be needed to investigate muscle strength, endurance, and contractile performance *in vivo*.

We also examined the effects of TH activity under *Thrap3* KO conditions by administering T3. We found that genes upregulated by T3 treatment in the Ctrl group but unchanged in the KO group included those involved in the AMPK pathway and the insulin signaling pathway. Indeed, previous studies have shown that T3 administration enhances AMPK pathway activity not only in skeletal muscle but also in several other organs [45–47]. In addition, we observed increased expression of *Akt2* and *Rptor*, key components of the mTOR pathway, which plays an essential role in muscle protein synthesis. Furthermore, genes related to cardiac muscle contraction were also upregulated by T3 treatment, consistent with previous reports indicating that excessive T3 levels can cause symptoms such as tachycardia and exercise intolerance [48,49]. These results suggest that many T3-inducible genes play important roles in muscle function and may interact with *Thrap3*. However, it should be noted that the serum used in this study already contained T3. Therefore, further experiments using T3-stripped serum will be necessary to more precisely characterize T3-inducible gene expression. Notably, previous studies using T3-depleted serum followed by T3 re-administration demonstrated increased *Vegfa* expression [24], which was not observed in our study. This discrepancy may be due to the presence of T3 in the serum used here, which could have weakened the effect of additional T3 treatment after myogenic differentiation.

## Supporting information

S1 Fig GEO dataset GSE469: microarray data of cardiotoxin-injured skeletal muscle. (TIFF)

S2 Fig Effects of *Thrap3* deletion on mitochondrial gene expression. mRNA expression levels of mitochondrial genes (*mt-Co3*, *mt-Atp6*, *mt-Nd3*, *mt-Nd4*) and nuclear-encoded mitochondrial genes (*Cox6c*, *Ndufs2*) in Ctrl and KO cells, as determined by qRT-PCR (n = 4/group).
(TIFF)

S3 Fig To exclude off-target effects, An independent *Thrap3* gRNA produced a second KO line. (A) Representative western blot and quantification of THRAP3 protein expression in Ctrl and KO cells at day 6 post-differentiation (n = 3/group). (B) mRNA expression levels of *Thrap3* in Ctrl and KO cells at day 6 post-differentiation (n = 4/group). (C) mRNA expression levels of *Myod1*, *Mef2c*, *Myog*, *Mybpc2*, *Myh1*, *Myh4*, and *Myh7* in Ctrl and KO cells at day 6 post-differentiation, as determined by qRT-PCR (n = 4/group).
(TIFF)

S4 Fig Heatmap of the top 100 most differentially expressed genes between *Thrap3* Ctrl and KO cells at day 7 post-differentiation. (TIFF)

S5 Fig Heatmap of genes involved in cell development and cell proliferation. (TIFF)

S6 Fig RNA-seq analysis of *Thrap3* KO cells with T3 treatment. (A) PCA of RNA-seq data from *Thrap3* KO cells with (T3+) and without (T3-) T3 treatment (n = 3/group). (B) KEGG pathway analysis of DEGs downregulated by T3 addition in *Thrap3* KO C2C12 cells. (C) KEGG pathway analysis of DEGs upregulated by T3 addition in *Thrap3* KO C2C12 cells.
(TIFF)

S7 Fig qRT-PCR analysis of an independently generated *Thrap3*-KO cell line treated with T3, performed to exclude potential off-target effects. (TIFF)

S8 Fig TPM values of *Bach1* obtained from RNA-seq analysis (n = 3/group). (TIFF)

S9 Fig Schematic diagram of the excitation-contraction (E-C) coupling mechanism in skeletal muscle. (TIFF)

S1 Raw images Raw data images of the original western blot images. (TIFF)
