# Supplementary figures and images for: Functional role of THRAP3 in modulating thyroid hormone–mediated gene networks in C2C12 myotubes
Source: PLoS One. 2026 Jan 22;21(1):e0341353. doi: 10.1371/journal.pone.0341353 (PMC12826480; doi:10.1371/journal.pone.0341353)

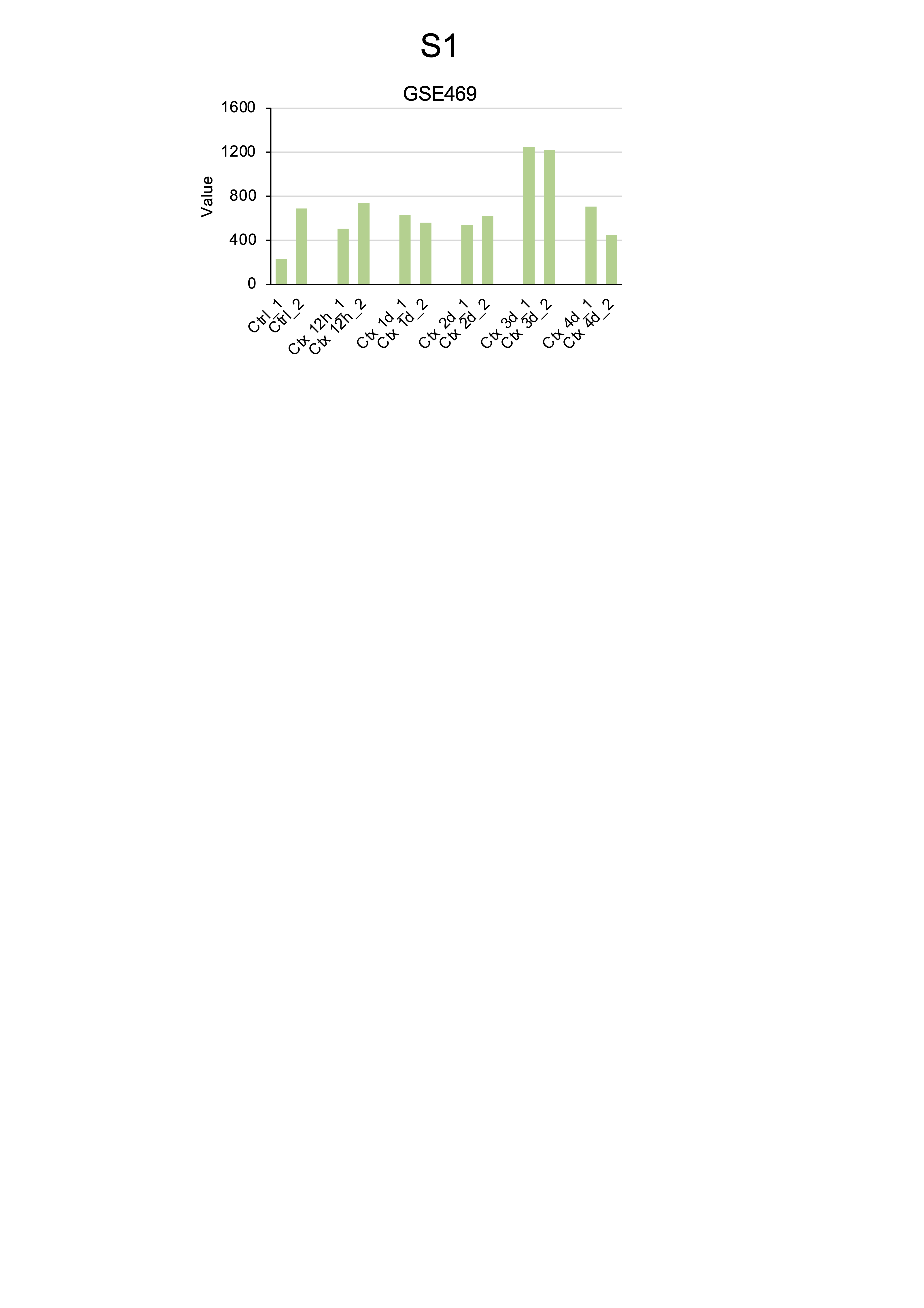

Supplement: S1 Fig — (TIFF) [file pone.0341353.s001.tiff]

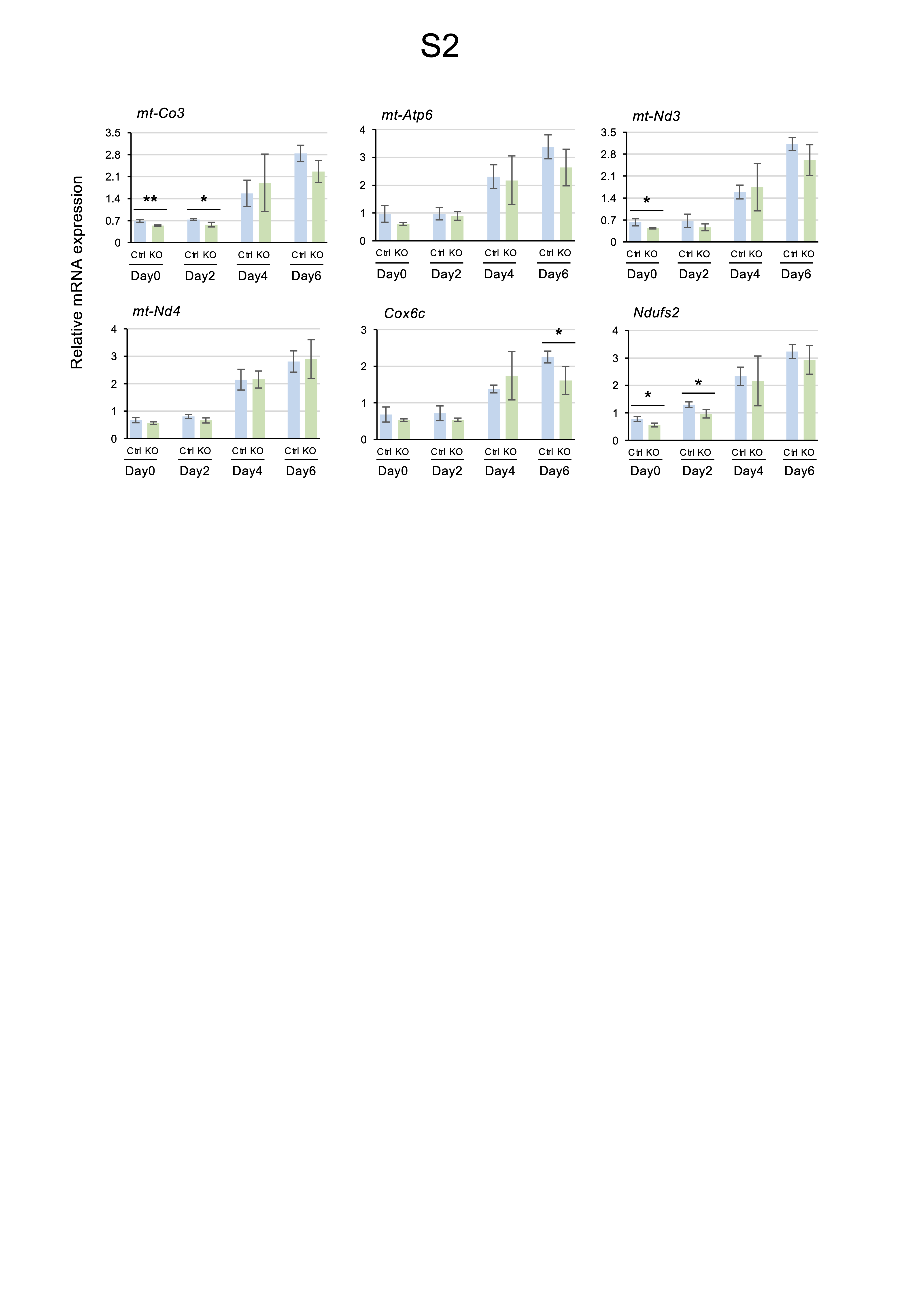

Supplement: S2 Fig — mRNA expression levels of mitochondrial genes (mt-Co3, mt-Atp6, mt-Nd3, mt-Nd4) and nuclear-encoded mitochondrial genes (Cox6c, Ndufs2) in Ctrl and KO cells, as determined by qRT-PCR (n = 4/group). (TIFF) [file pone.0341353.s002.tiff]

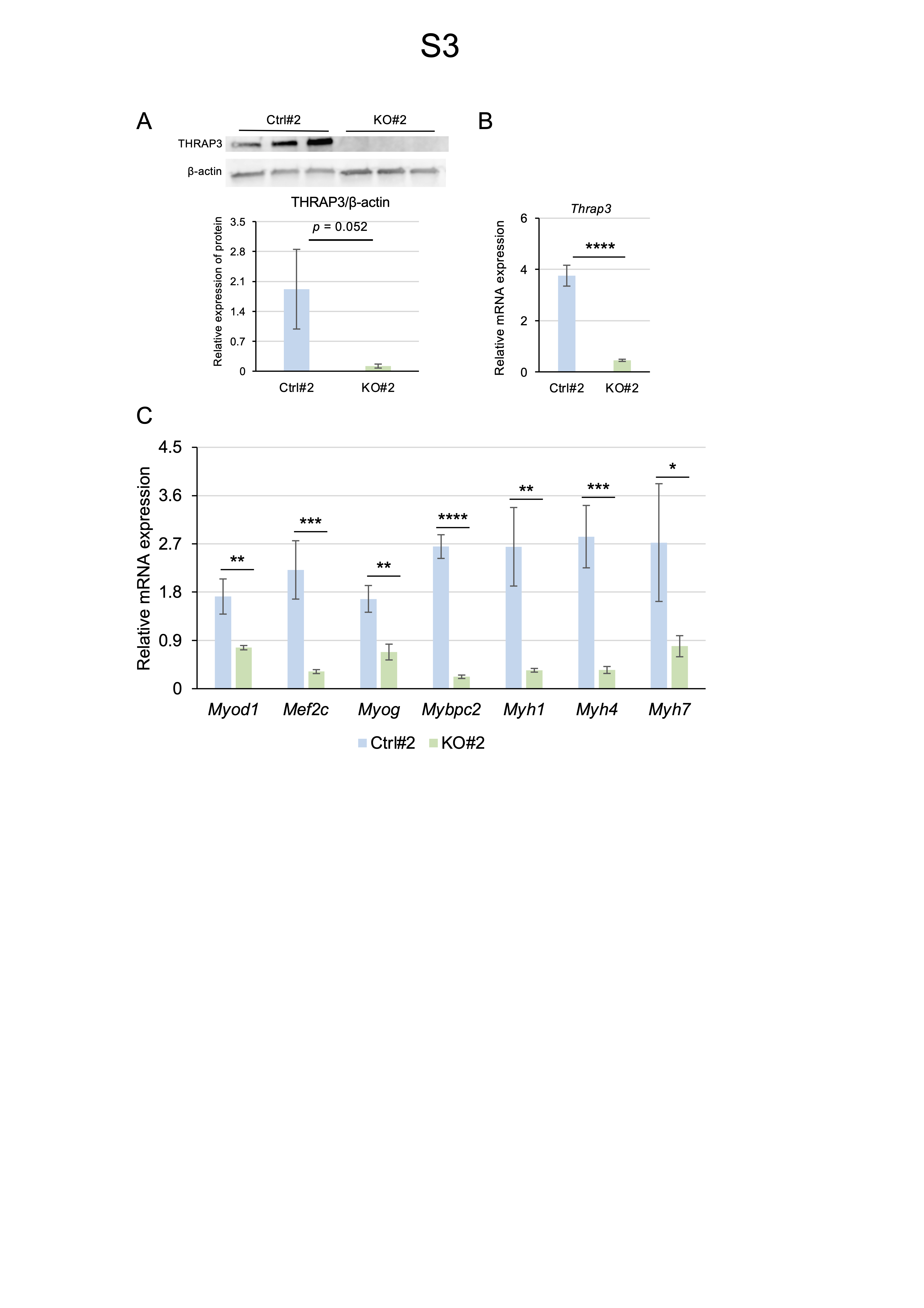

Supplement: S3 Fig — (A) Representative western blot and quantification of THRAP3 protein expression in Ctrl and KO cells at day 6 post-differentiation (n = 3/group). (B) mRNA expression levels of Thrap3 in Ctrl and KO cells at day 6 post-differentiation (n = 4/group). (C) mRNA expression levels of Myod1, Mef2c, Myog, Mybpc2, Myh1, Myh4, and Myh7 in Ctrl and KO cells at day 6 post-differentiation, as determined by qRT-PCR (n = 4/group). (TIFF) [file pone.0341353.s003.tiff]

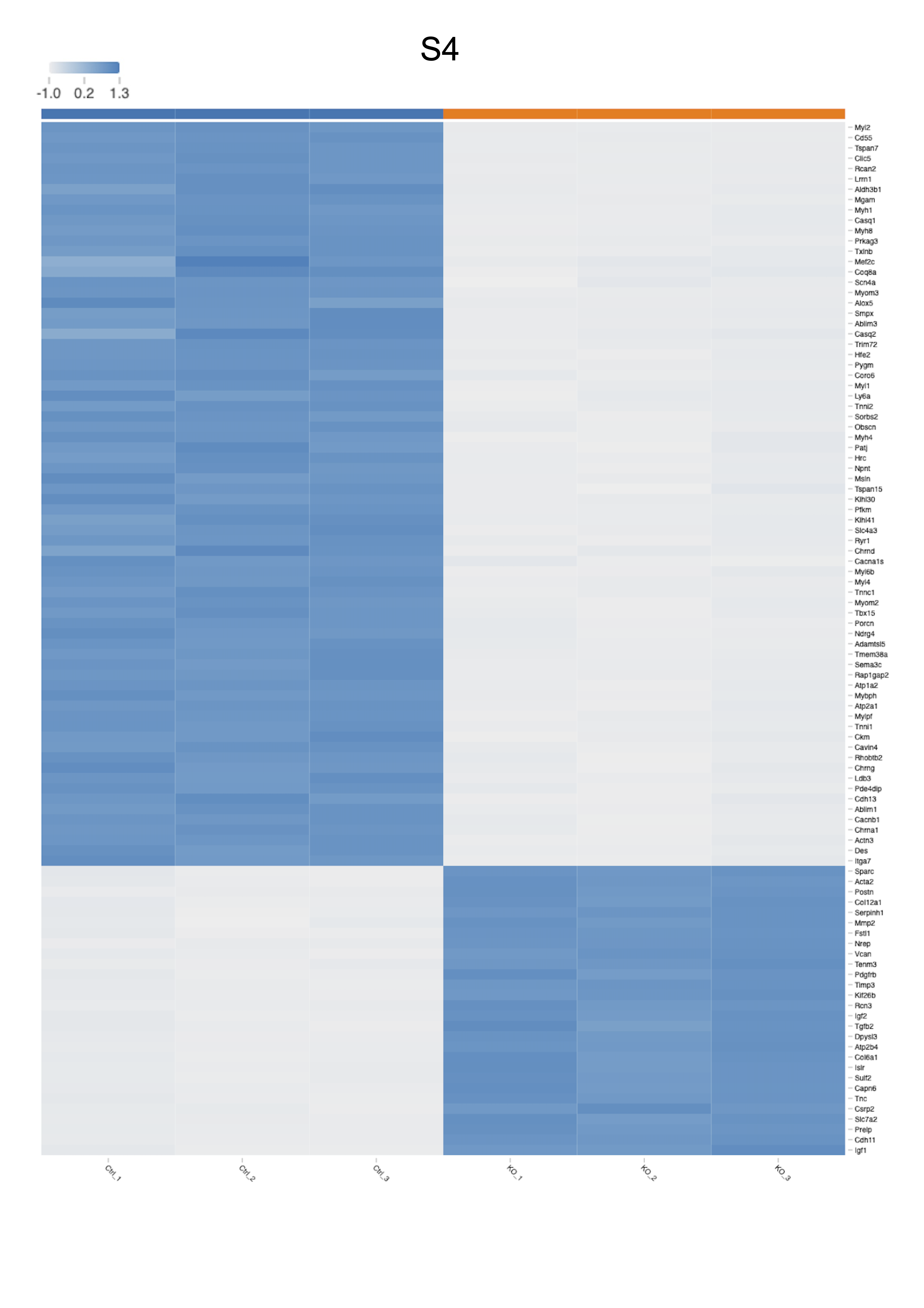

Supplement: S4 Fig — (TIFF) [file pone.0341353.s004.tiff]

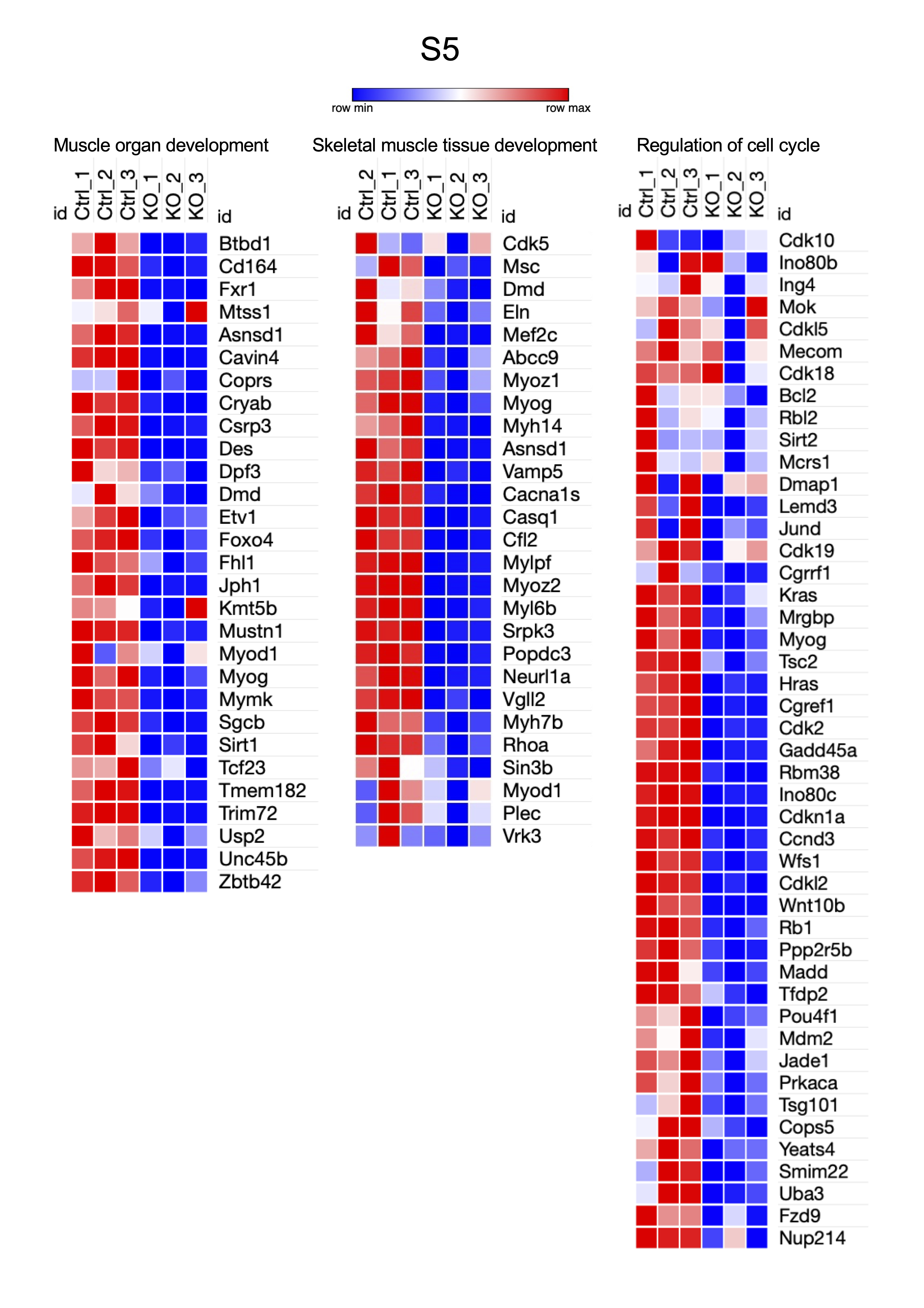

Supplement: S5 Fig — (TIFF) [file pone.0341353.s005.tiff]

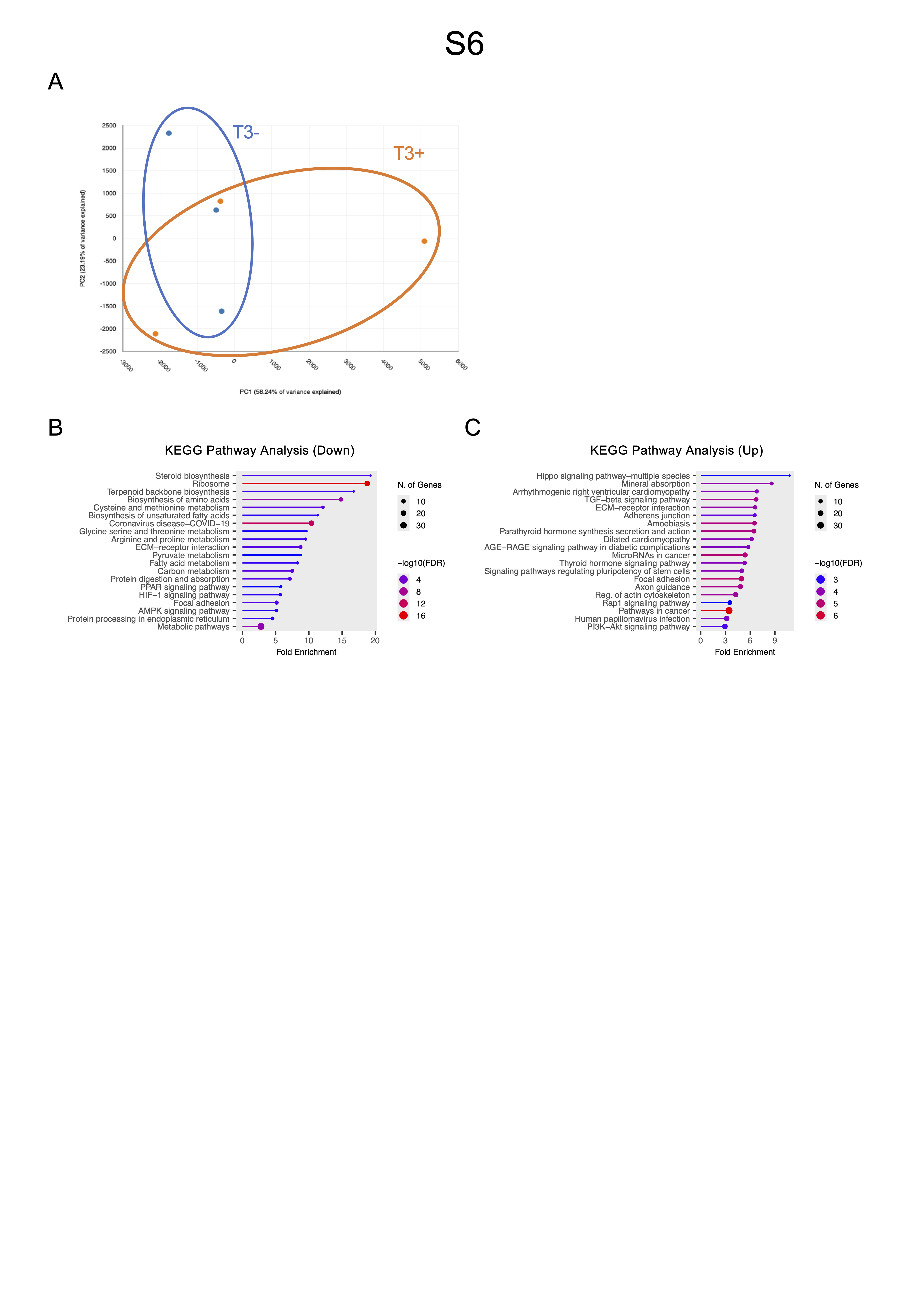

Supplement: S6 Fig — (A) PCA of RNA-seq data from Thrap3 KO cells with (T3+) and without (T3-) T3 treatment (n = 3/group). (B) KEGG pathway analysis of DEGs downregulated by T3 addition in Thrap3 KO C2C12 cells. (C) KEGG pathway analysis of DEGs upregulated by T3 addition in Thrap3 KO C2C12 cells. (TIFF) [file pone.0341353.s006.tiff]

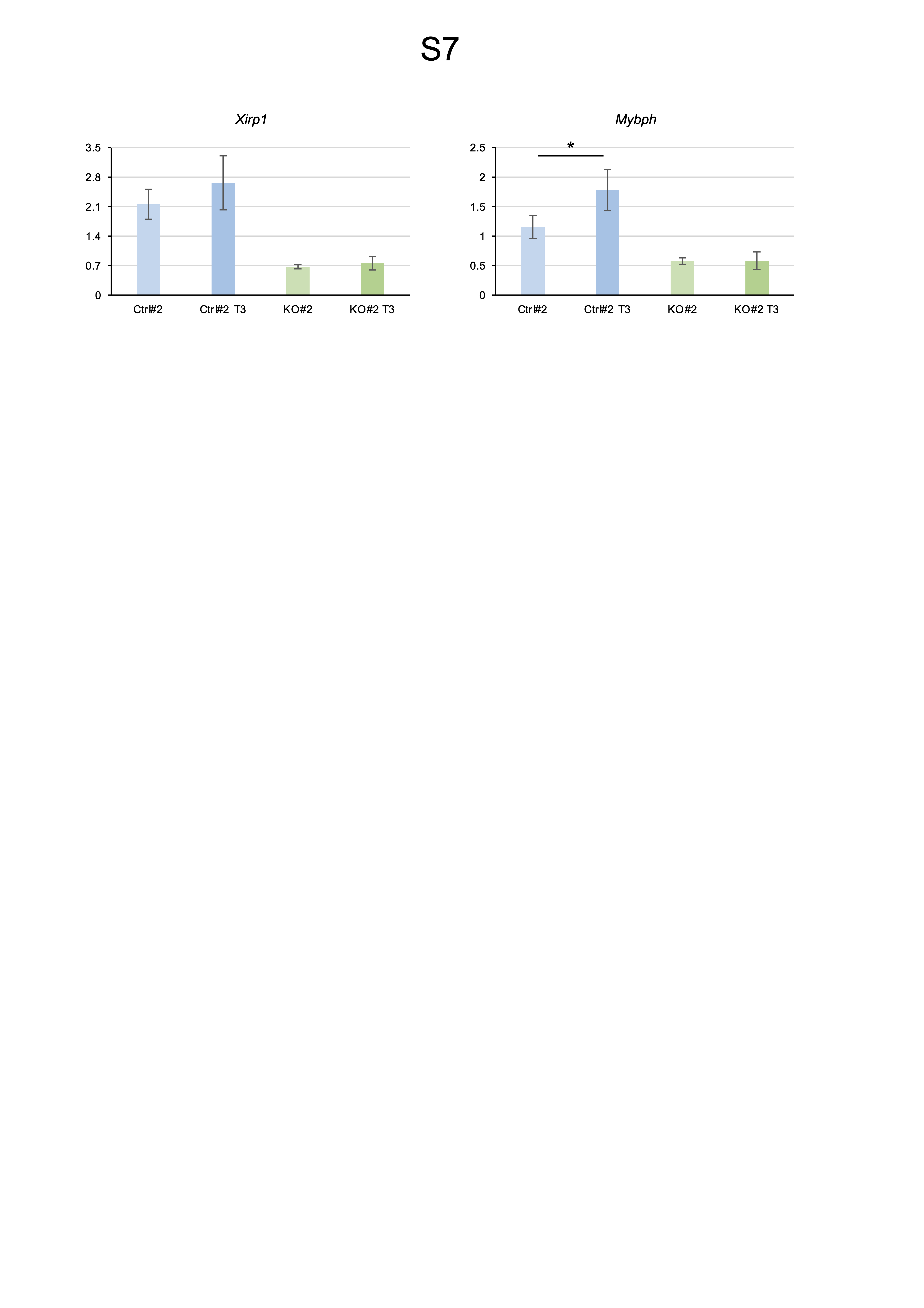

Supplement: S7 Fig — (TIFF) [file pone.0341353.s007.tiff]

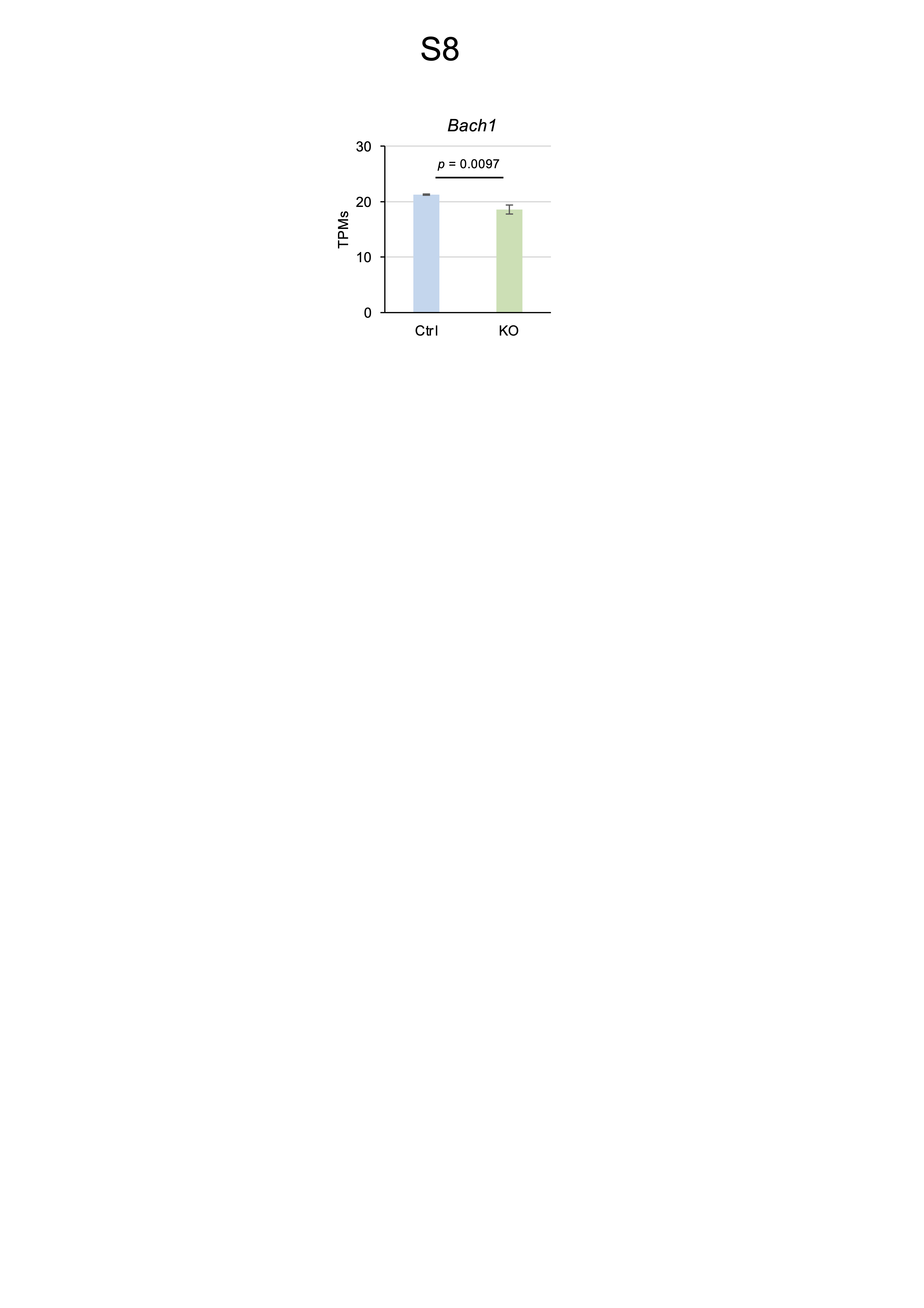

Supplement: S8 Fig — (TIFF) [file pone.0341353.s008.tiff]

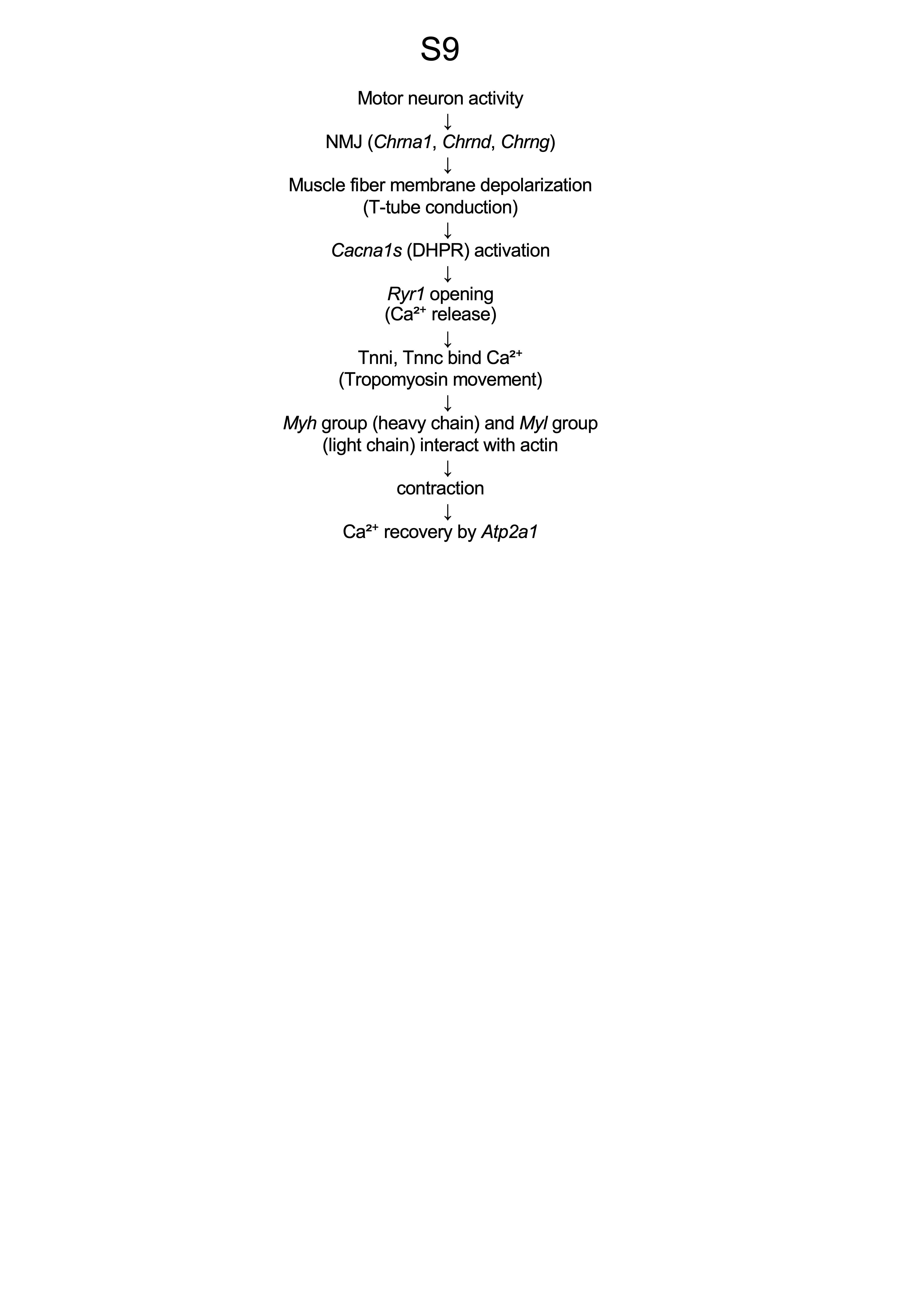

Supplement: S9 Fig — (TIFF) [file pone.0341353.s009.tiff]

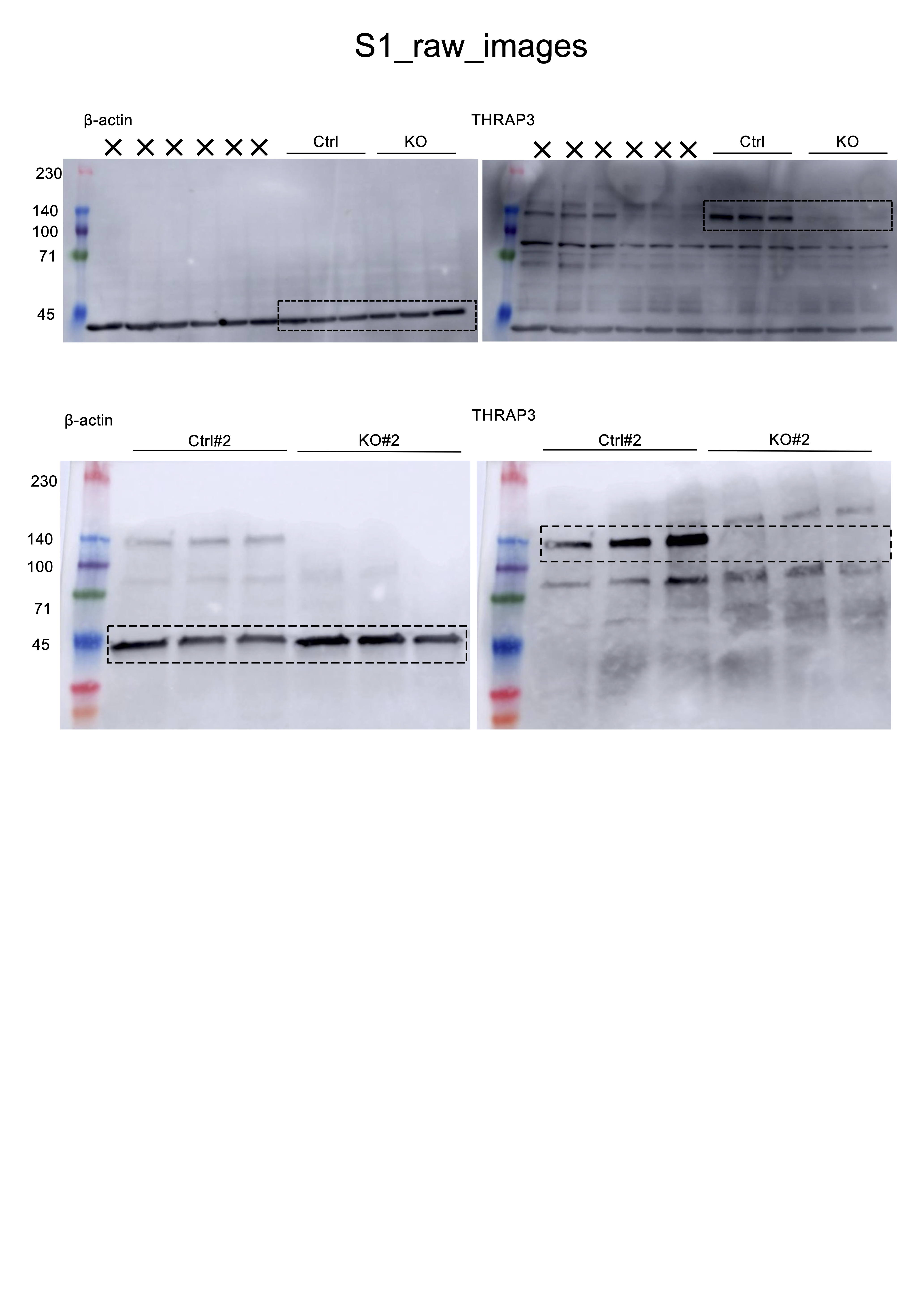

Supplement: S1 Raw images — (TIFF) [file pone.0341353.s010.tiff]
